# Obstruction of the Bowel on an Inter-sigmoid Hernia: A Rare Cause of Occlusion

**DOI:** 10.7759/cureus.77996

**Published:** 2025-01-26

**Authors:** Haitam Soussan, Soufiane Taibi, Abdelali Guellil, Rachid Jabi, Mohammed Bouziane

**Affiliations:** 1 Department of General Surgery, Mohammed VI University Hospital, Faculty of Medicine and Pharmacy, Laboratory of Anatomy, Microsurgery and Surgery Experimental and Medical Simulation (LAMCESM) Mohammed 1st University, Oujda, MAR; 2 Visceral Surgery and Digestive Oncology A, Mohammed VI University Hospital, Oujda, MAR; 3 Visceral Surgery, Mohamed VI University Hospital, Oujda, MAR; 4 General Surgery A, Mohammed VI University Hospital, Oujda, MAR

**Keywords:** internal hernia, intersigmoid fossa, intersigmoid hernia, intestinal obstruction, mesocolic hernia

## Abstract

Inter-sigmoid hernia is a rare condition characterized by the protrusion of the small intestine into the inter-sigmoid fossa. The inter-sigmoid fossa, also known as the inter-sigmoid recess, is a funnel-shaped peritoneal pouch of variable size, located between the two mesenteric roots of the parietal margin of the meso-sigmoid.

The diagnosis of inter-sigmoid hernia is often delayed due to nonspecific clinical symptoms, which frequently leads to a postponement of surgical intervention. Preoperative detection on CT imaging can be challenging, and the diagnosis is typically confirmed during urgent surgical exploration.

We report the case of a 63-year-old man who presented to the emergency room with abdominal pain. The physical examination and radiological findings were suggestive of an intestinal occlusion over an internal hernia. Given these findings, an emergency laparotomy was performed, confirming the diagnosis of an inter-sigmoid internal hernia with incarceration of a distended segment of the small intestine.

## Introduction

Internal hernias are characterized by the protrusion of abdominal viscera, most commonly the small bowel, through an intra-abdominal or retroperitoneal defect [1]. They represent a rare cause of acute intestinal obstruction (AIO), accounting for 0.2% to 5.8% of AIO cases reported in the literature [2].

Internal hernias present a wide range of anatomical presentations, some of which are extremely rare [2]. The intersigmoid hernia arises from a congenital fossa formed by the fusion of the two roots of the sigmoid mesocolon. It extends superiorly, behind the descending mesocolon, and is located between the lumbar spine [3].

Based on a clinical observation and a review of existing literature, the aim of this study is to explore the diagnostic challenges and therapeutic strategies associated with this uncommon condition.

## Case presentation

**Figure 1:**
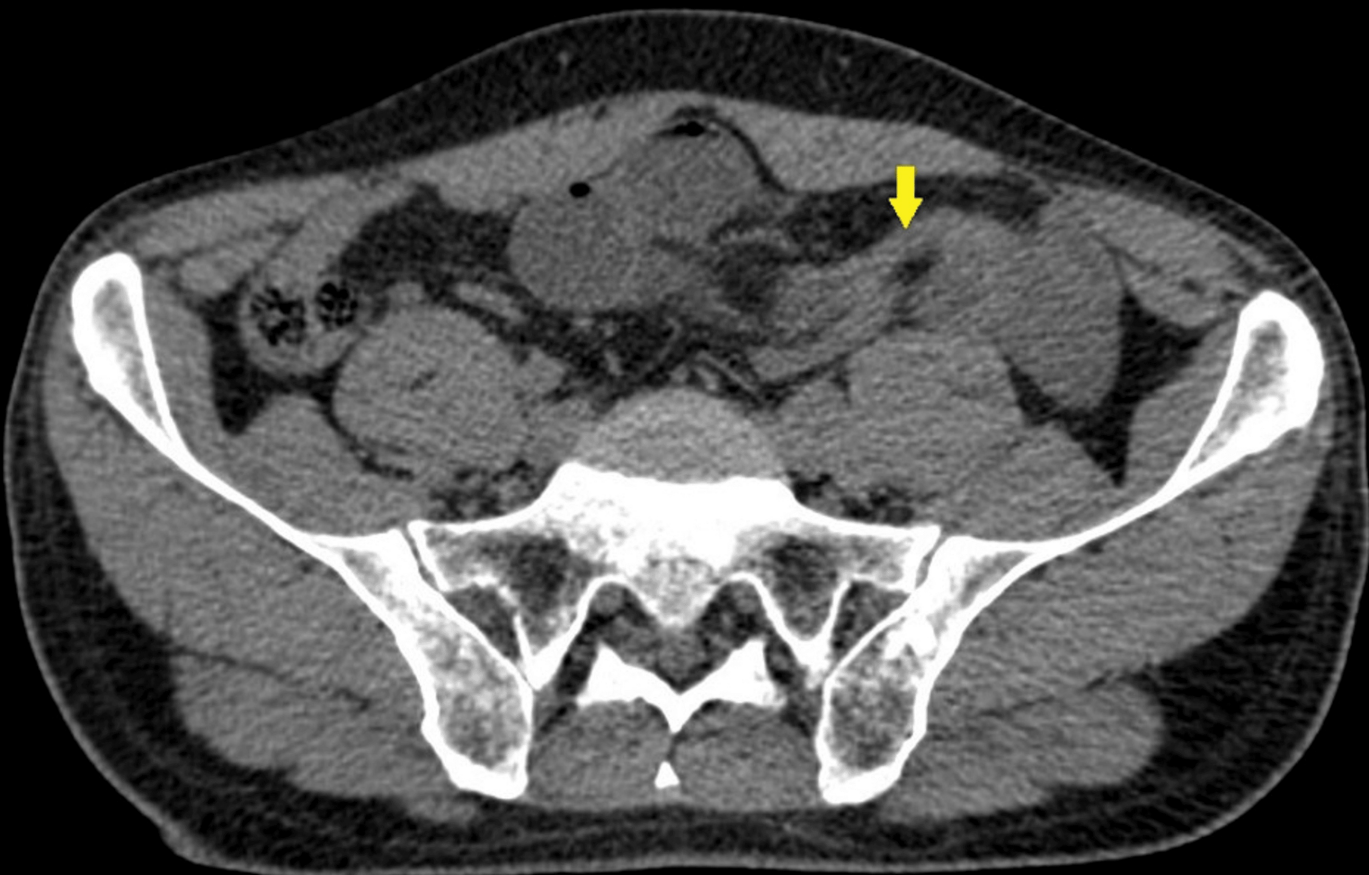
CT scan showing the transition zone in the left iliac fossa in axial section Yellow arrow: Zone of transition

**Figure 2:**
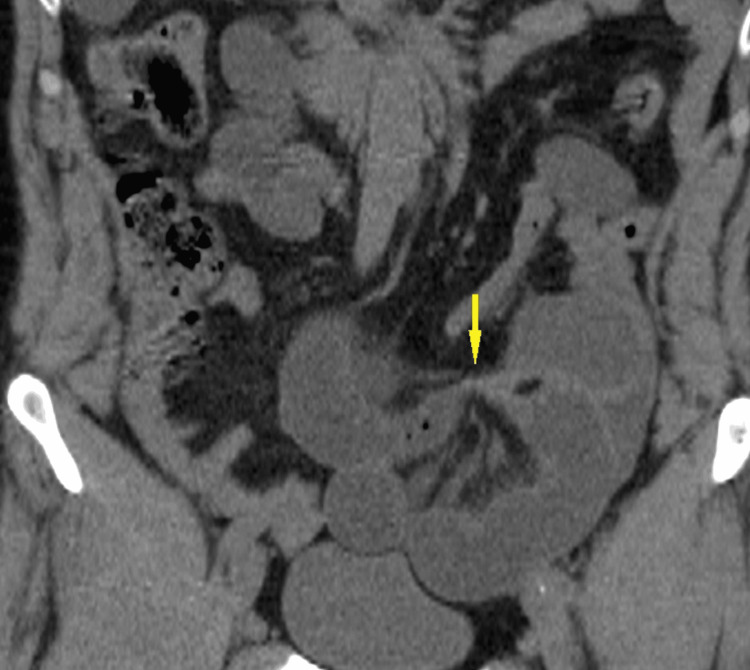
Coronal CT scan showing encapsulation and arrangements of distended small bowel loops withing abnormal sac in left iliac fossa giving -C- shape for the distended segment, suggesting an internal inter-sigmoid hernia. Yellow arrow: Zone of transition

A 63-year-old male with no significant medical history presented to the emergency department with abdominal pain, accompanied by cessation of flatus and stool, as well as vomiting. Upon further questioning, the patient reported previous similar episodes that resolved spontaneously. Clinical examination revealed abdominal distension and tenderness upon palpation, with no abnormalities noted at the hernial orifices. Laboratory tests were unremarkable, with no evidence of electrolyte imbalances. Abdominal X-ray in standing position showed a thin bowel loop with hydro air levels. A subsequent CT scan revealed distension of the cecum, likely due to an inter-sigmoid hernia, with signs of edematous congestion of the mesentery and a small amount of free fluid (Figures 1, 2).

**Figure 3:**
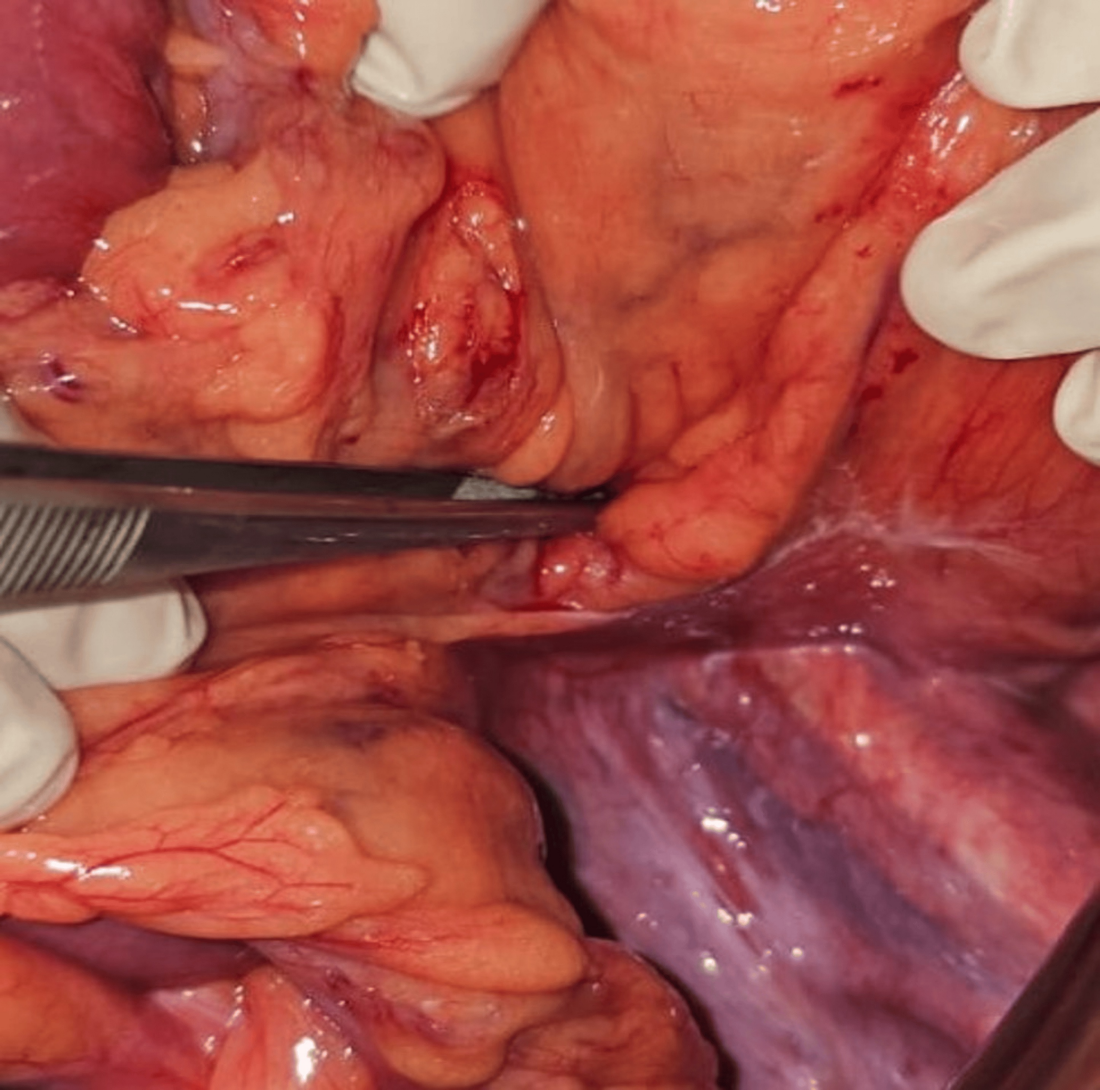
Intraoperative view showing the the inter-sigmoid fossa.

An emergency laparotomy was performed due to the lack of laparoscopic technical facilities. The diagnosis was confirmed as an inter-sigmoid internal hernia with incarceration of the distended and infarcted segment of the small intestine. The incarcerated bowel was released, and the hernial defect was closed with separate sutures, ensuring the preservation of the mesocolonic vessels (Figure 3).

Given the absence of prior abdominal surgery or trauma, the hernia was considered to be of congenital origin. Postoperative recovery was uneventful, and the patient was discharged on the second day after surgery.

## Discussion

Internal hernias are rare and typically classified into two categories: congenital and iatrogenic (postoperative) hernias. Inter-sigmoid hernias, along with para-duodenal and para-caecal hernias, are characterized by their occurrence through a normal or so-called "paranormal" peritoneal opening [1].

**Figure 4:**
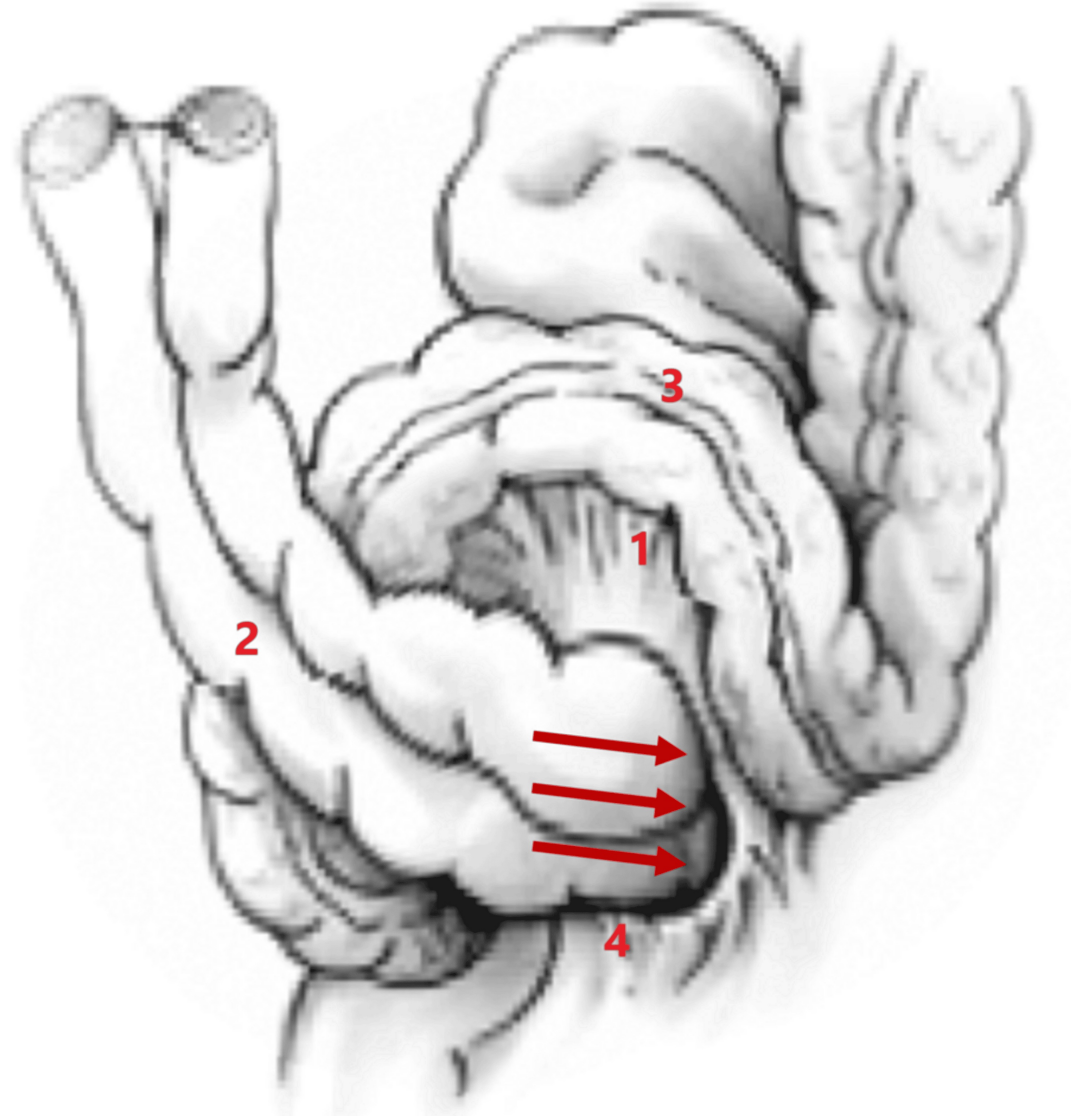
Explanatory diagram of an inter-sigmoid hernia. 1: Mesentery of sigmoid colon 2: Incarcerated small intestine 3: Sigmoid colon 4: Inter-sigmoid fossa Image modified from Chiarini et al. [6] under CC BY 4.0

Inter-sigmoid hernias are rare, accounting for approximately 6% of all internal hernias [4,5]. In fact, only 124 cases have been reported between 1964 and 2019, according to a study by Chiarini et al. [6]. This type of hernia develops in a cul-de-sac known as the intersigmoid fossa. This is a V-shaped peritoneal structure of variable size located between the two roots of the parietal margin of the sigmoid mesocolon (Figure 4) [6]. This fossa is the fourth most common cause of internal hernia, after para-duodenal, para-caecal and transmesenteric hernias [5].​

Internal hernias are often incidentally discovered during imaging for other conditions or, less commonly, present as bowel obstruction without clear clinical signs [6]. This often leads to a delayed diagnosis, which increases the risk of necrosis and ischemia due to vascular strangulation.

CT imaging plays a critical role in diagnosis of acute intestinal obstruction, identifying its underlying cause and associated complications. It detects the internal hernias in 77% of cases, with a sensitivity of 63% and specificity of 76% [7].

The possibility of an internal hernia should be considered in young patients with bowel obstruction and no history of surgery or abdominal trauma, particularly when there is a history of sub-occlusive episodes that resolve spontaneously. Despite these clinical clues, preoperative diagnosis of inter-sigmoid hernias remains challenging [6]. However, advances in CT scan and magnetic resonance imaging have significantly enhanced diagnostic accuracy.

Once the diagnosis is confirmed, emergency surgery is crucial. The surgical procedure typically involves reduction of the incarcerated viscera, with or without performing bowel resection, if necessary, followed by closure of the peritoneal fossa or the abnormal orifice when feasible [7].

While laparoscopic surgery is preferred due to its several advantages over traditional open surgery, it may become challenging and risky in cases of significant small bowel distension [8].

## Conclusions

Hernias of the inter-sigmoid fossa present as a rare cause of acute intestinal obstruction; the clinical variability makes the diagnosis difficult. In the context of a young individual experiencing symptoms of bowel obstruction without a history of abdomino-pelvic surgery, suspicion of an internal hernia becomes significant.
